# Biliary adverse events in acromegaly during somatostatin receptor ligands: predictors of onset and response to ursodeoxycholic acid treatment

**DOI:** 10.1007/s11102-020-01102-7

**Published:** 2020-11-09

**Authors:** N. Prencipe, C. Bona, D. Cuboni, M. Parasiliti-Caprino, A. M. Berton, L. M. Fenoglio, V. Gasco, E. Ghigo, S. Grottoli

**Affiliations:** 1 Division of Endocrinology, Diabetology and Metabolism, Department of Medical Science, University of Turin, Corso Dogliotti 14, 10126 Turin, Italy; 2 Division of Internal Medicine, Santa Croce and Carle General Teaching Hospital, Cuneo, Italy

**Keywords:** Gallstone, Octreotide, Pasireotide, Growth hormone, Sludge, Lanreotide

## Abstract

**Purpose:**

Somatostatin receptor ligands (SRL) are the first-line medical treatment for acromegaly. Gallbladder alterations are one of most important SRL side effect, but according to some authors growth hormone hypersecretion itself is a risk factor for gallstones. This single center, longitudinal retrospective study evaluated the incidence and the predictors of biliary adverse events (BAE) in acromegaly during SRL therapy and their response to ursodeoxycholic acid (UDCA).

**Methods:**

91 acromegaly patients with indication to SRL were enrolled. Evaluations of acromegaly activity (GH, IGF-I, IGF-I/ULN) and metabolic profile were collected before starting treatment, yearly during follow-up and at BAE onset. In patients developing BAE we searched for predictors of UDCA effectiveness.

**Results:**

61.5% of patients developed BAE (58.9% cholelithiasis; 41.1% only sludge). IGF-I and IGF-I/ULN proved to be positive predictor of BAE, which occur about 5 years after SRL starting. None of metabolic markers proved to be associated with BAE. Only five patients (5.5%) underwent cholecystectomy for symptomatic cholelithiasis. 71% of patients started UDCA treatment, achieving regression of BAE in 60% of cases (88% in patients developing only sludge and 30% in patients affected by cholelithiasis, p < 0.001). BMI and obesity were negative predictors of UDCA efficacy. In 50% of the subjects BAE resolved after 36 months of therapy with a lower rate if cholelithiasis was present.

**Conclusion:**

Biliary stone disease is a frequent SRL adverse event, although it is often symptomless. Ultrasound follow-up mainly in the first 5 years of therapy, early UDCA starting and proper lifestyle represent a valid strategy in their detection and management.

## Introduction

Somatostatin receptor ligands (SRL) are the first-line medical treatment in acromegaly disease because of their action in inhibiting pituitary growth hormone (GH) secretion. In acromegaly patients, SRL represent long-term treatment, sometimes even lifelong. SRL treatment is well tolerated and characterized by a low incidence of adverse events (AEs). In particular, in the Literature biliary complications rate ranges between 3.6 and 56% of patients, regardless of the molecule type [1].

Cholesterol gallstones form when the cholesterol concentration in bile exceeds the ability of bile to hold it in solution, so that crystals form and grow as stones. Gallbladder sludge, thickened gallbladder mucoprotein with tiny entrapped cholesterol crystals, is thought to be the usual precursor of gallstones. Sludge can sometimes cause biliary pain, cholecystitis, or acute pancreatitis, but sludge may also resolve without treatment [2].

Physiologically, somatostatin inhibits the secretion of bile salts, promotes sodium and water absorption by the gallbladder (increasing bile concentration) and causes a reduction in the post-prandial release of cholecystokinin (CCK) [3]. The CCK, through cholinergic receptors activation of the myenteric plexus, promotes gallbladder contraction and therefore its emptying. Thus, during SRL, lower CCK levels causes a motility defect of the gallbladder, consequent biliary stasis and stones development [4]. Hofmann also documented a reduction in postprandial sphincter of Oddi relaxation, another factor favouring biliary stasis [5].

One of the first studies on the association between biliary stones and SRL in acromegaly patients reported that 23% of patients developed gallbladder stones and 20.6% biliary sludge; SRL dose did not reveal a determining factor and most of the events occurred within the first year of treatment [6]. In a more recent article cholelithiasis onset was assessed after 1–2 years of SSA therapy [1], most patients who develop a biliary AE (BAE), are asymptomatic and less than 1% needed cholecystectomy [1, 7].

Also in neuroendocrine neoplasms (NET) SSA are first line medical treatment but in this setting BAEs incidence seems lower (36.6%) and belated (after 36.7 months of treatment), compared to acromegaly patients [8]. Because of the disagreement about the BSD risk factors, it is not feasible nowadays to establish a univocal strategy to manage these AE. In the general population, multiple genetic and exogenous risk factors have been identified. A Swedish study conducted on a large number of twins showed that 25% of the risk is genetically determined [9]. Instead, as for environmental factors, obesity, metabolic syndrome and type 2 diabetes mellitus (DM) seem to be the main determinants [10–12]. Many studies investigated the association between cholelithiasis and lipid profile, with conflicting results but confirming high non-HDL cholesterol levels as the only recognized risk factor [13]. In SRL-treated acromegaly patients, as well as in the general population, obesity and dyslipidaemia represent important risk factors [1, 7].

According to some authors, 16–26% of acromegalic patients presents biliary stones even in the absence of treatment [14–16], suggesting that acromegaly itself may be a risk factor for gallstones. However, a retrospective study on 459 SRL treated patients did not show any difference between acromegaly patients and individuals with SRL therapy for different diagnosis [17].

The European Association for the Study of the Liver (EASL) Guidelines consider SRL treated patients as a high-risk population and recommend both ursodeoxycholic acid (UDCA) treatment and an eventual prophylactic cholecystectomy. Anyway, these recommendations are weak and based on few small studies [18].

The aim of this study was to analyse the incidence of BAEs and the predictive factors for the BSD onset in a large population of acromegaly patients treated with long acting release (LAR) SRL. The secondary outcome was to evaluate the incidence and the predictors of recovery during UDCA therapy.

## Materials and methods

### Subjects

**Fig. 1:**
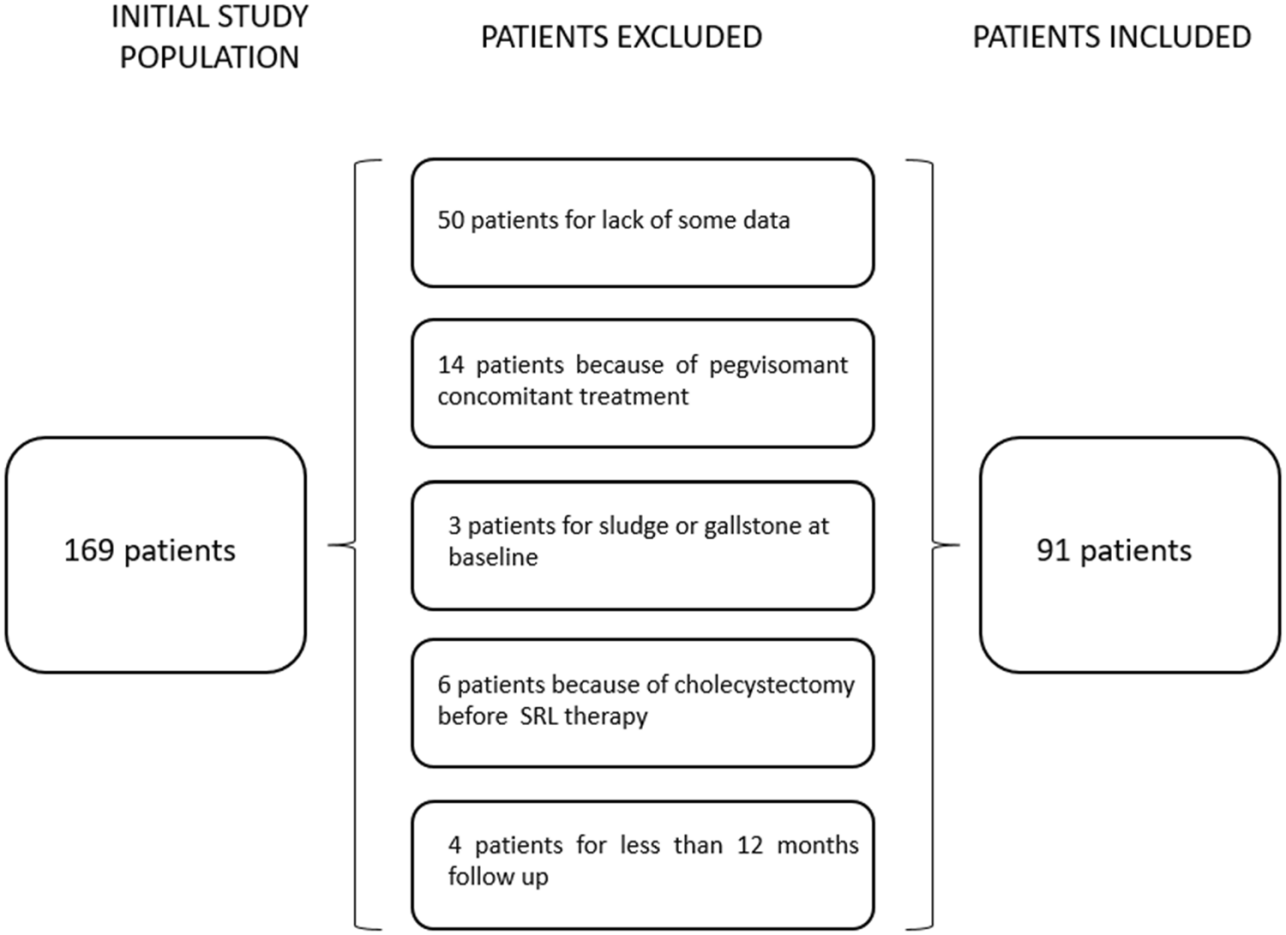
Diagram of initial study population, excluded and included patients. *SRL* somatostin receptor ligand

Data of all acromegaly patients, treated with SRL for at least 1 year, referred to the Division of Endocrinology, Diabetology and Metabolism, “Città della Salute e della Scienza” Hospital of Turin Italy, were collected retrospectively from prospective registry. As shown in the inclusion diagram (Fig. 1), we included in the analysis only patients with all available data for each year between the start of SRL treatment and the adverse event onset or last follow up. Only patients with pre-treatment ultrasound showing no evidence of biliary diseases were included in the study. Exclusion criteria were: partial or absent availability of data on basal status and follow up and the absence of patient consent.

For each patient, we recorded clinical and demographic features at diagnosis and before SRL starting. We also collected hormonal (GH and IGF-I), metabolic variables (ALT, AST, GGT, fasting glucose and HbA1c levels), the presence of hypertriglyceridemia and diabetes mellitus (DM), treatment features (SRL molecule, dose and duration of treatment) and abdominal ultrasound data for each year of SRL treatment. Based on abdominal ultrasound, we collected information relative to sludge or gallstones presence, symptomatic cholelithiasis onset, cholecystectomy and UDCA therapy. We calculated also the IGF-I/upper limit of normal ratio (IGF-I/ULN).

The study was conducted in accordance with guidelines in the Declaration of Helsinki. Approved from the Ethical Committee of “Città della Salute e della Scienza” University Hospital of Turin was obtained and all patients provided their written informed consent.

### Statistics

Baseline characteristics of all patients included in the analysis are summarized using median and interquartile range (IQR) for continuous data (or mean and standard deviation when specified) and rate and percent values for binary and categorical data. Between-group differences in personal and clinical features at diagnosis were evaluated by the Student’s *t* test, the Mann–Whitney U test, the ANOVA and the Kruskal–Wallis test for continuous variables and the Chi square test or Fisher’s exact test for categorical variables, where appropriate considering the normality with Shapiro–Wilk test and number of independent groups.

The cumulative incidence of BAE was estimated using the Kaplan–Meier method. Statistical significance (p < 0.05) of differences in the cumulative incidence of BAE between groups was tested using the log-rank test for homogeneity.

The observation period for time to BAE started with the day of SRL therapy starting until the BAE development (failures), or until the last follow-up visit (censoring). A Cox proportional hazard model was employed to estimate the crude and the multivariable-adjusted hazard ratios (HRs) with 95% confidence intervals (CIs) and to evaluate possible predictors of BAE development.

The effect of the following selected factors, potentially associated with BAE development, was considered in the univariate models: baseline GH, IGF-I, IGF-I/ULN, age, glucose and HbA1c; presence of diabetes and hypertriglyceridemia at baseline, gender, SRL molecule and SRL monthly dose.

Also the cumulative incidence of BAE regression was estimated using the Kaplan–Meier method and statistical significance (p < 0.05) of differences in the cumulative incidence of BAE regression between groups G_CH_ and G_SL_ was tested using the log-rank test for homogeneity. The observation period for time to BAE regression started with the day of BAE development, which overlaps with the day of UDCA therapy starting, until the time of BAE regression (failures), or until the last follow-up visit (censoring). A Cox proportional hazard model was employed to estimate the crude and the multivariable-adjusted HRs with 95% CIs and to evaluate possible predictors of BAE regression. The effect of the following selected factors, potentially associated with BAE regression, was considered in the univariate models: cholelithiasis development, GH, IGF-I, IGF-I/ULN, glucose, HbA1c, triglycerides, LDL, HDL, non-HDL, GGT, BMI and age at BAE onset; presence of diabetes and hypertriglyceridemia at BAE onset, gender, SRL molecule and SRL monthly dose.

Patients treated both with first generation SRL and pasireotide, came out of the statistical analysis when they stopped first generation SRL and started pasireotide.

In all the models, the proportional hazard assumptions were also verified by graphical checks and formal tests based on Schoenfeld residuals. Statistical analysis was performed using MedCalc™, version 18.11.3.

## Results

### Clinical features at baseline

We enrolled 91 acromegaly patients (33 males and 58 females), in follow-up for a median of 132 months (range 12–444 months) and under SRL therapy for 84 (12–252) months. Eighty-eight (97%) had a pituitary adenoma (71 macroadenoma; 17 microadenoma). Among the three remaining patients, one had a GHRH secreting pulmonary NET and two had an empty sella and it was not possible to identify a pituitary adenoma. Mean age at baseline was 48.9 ± 15 years; median GH before starting SRL was 6.1 (3.7–15.0) ng/mL, median IGF-I was 689 (460–972) ng/mL and median IGF-I/ULN was 2.3 (1.6–3.3). Fifty-nine patients (65%) were treated with octreotide LAR and 32 (35%) with lanreotide autogel; 5 patients were subsequently shifted to pasireotide LAR because they revealed treatment resistant. Basally 12 patients (13%) had hypertriglyceridemia and 17 (19%) type 2 DM without gender differences; before SRL therapy, median fasting glucose was 92 (86–102) mg/dL and HbA1c was 40 (38–44) mmol/mol (Table 1).

**Table 1:** Patients features before somatostatin receptor ligands starting

	Overall data (n. 91)	G− group (n. 35)	G+ group (n. 56)	p value
Age (years) Mean ± SD	48.9 ± 15	50.2 ± 16.1	48.2 ± 14.5	NS
Macroadenoma n. (%)	71 (78)	27 (77.1)	44 (78.6)	NS
Microadenoma n. (%)	17 (18.7)	6 (17.1)	11 (19.6)	
Surgery n. (%)	46 (52)	17 (50)	29 (51.8)	NS
Radiotherapy n. (%)	11 (13)	4 (11.8)	7(12.5)	
Males n. (%)	33 (36)	9 (25.7)	24 (42.8)	NS
IGF-I (ng/mL) median IQR	689 (460–972)	651 (441.5–949.5)	719 (468.7–1058)	NS
GH (ng/mL) median IQR	6.1 (3.7–15)	5.7 (2.7–15.9)	6.3 (3.8–13.9)	NS
IGF-I/ULN median IQR	2.3 (1.6–3.3)	2 (1.6–3)	2.5 (1.6–3.5)	NS
Hypertrygliceridemia n. (%)	12 (13)	6 (17.6)	6 (10.7)	NS
Diabetes n. (%)	17 (19)	8 (24.2)	9 (16.1)	NS
Glycemia (mg/dL) median IQR	92 (86–102)	98 (85.5–107)	90 (86–99.5)	NS
HbA1c (mmol/mol) median IQR	40 (38–44)	42 (39.7–46)	40 (37–42.5)	**0.039**

Statistically significant value (p < 0.05) is given in bold

*ULN* upper limit of normal, *GH* growth hormone, *HbA1c* glycosylated hemoglobin, *CI* confidence interval, *IGF*-*I* insulin like growth factor I

Thirty-five individuals (39.5%) did not develop biliary adverse event (G− group) and 56 (61.5%) patients developed biliary adverse event (G+ group). Among the last group, we identified two further subgroups: G_CH_ (33 patients) who developed cholelithiasis and G_SL_ (23 subjects) who developed only sludge. Five patients also had symptomatic cholelithiasis and underwent cholecystectomy (4 in election and 1 patient in urgency).

### Biliary adverse events development

Demographic, clinical and biochemical features of G− and G+ groups are summarized in Table 1. No differences were detected in gender, age, metabolic and hormonal parameters, as well as associated comorbidities (obesity, DM and hypertriglyceridemia). The SRL treatment duration was longer in G− compared to G+ group (36, 12–252 vs 24.8, 12–144; p = 0.011). Octreotide and lanreotide treatment showed the same risk of complications and no differences in the prescribed drug rates were found in the two groups.

**Fig. 2:**
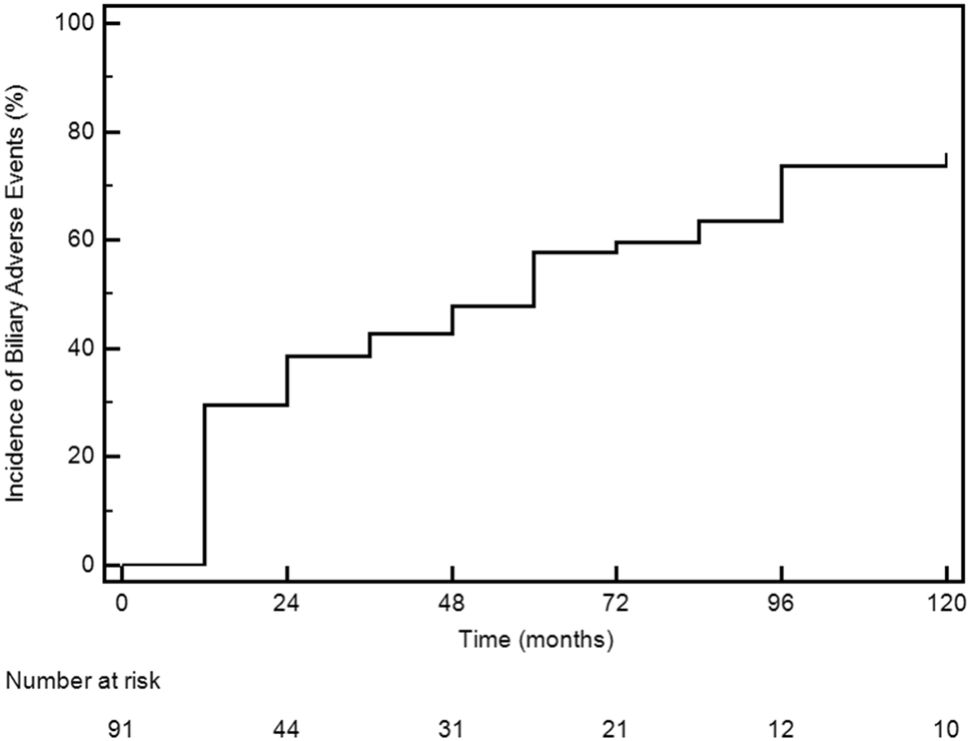
Kaplan–Meier curve for biliary adverse event (BAE) development

In Fig. 2, the Kaplan–Meier curve showed that the 50% of the subjects at risk developed a BAE within 5 years from SRL starting. In Cox model regression higher IGF-I levels (HR 1.001, 95% CI 1.000–1.0012; p = 0.032) and higher IGF-I/ULN ratio (HR 1.337, 95% CI 1.081–1.654; p = 0.008) before SRL starting proved to be the only predictors of BAE development. This result remained statistically significant also in the multivariate Cox regression models (Table 2). In G_CH_ group, 10 patients (30%) had a diagnosis of sludge prior to microlithiasis development, while in 23 subjects (70%) gallstones and sludge were detected simultaneously. No differences were detected in gender, age, metabolic and hormonal parameters, comorbidities and prescribed treatment between G_CH_ and G− group (data not showed).

**Table 2:** Multivariate models of COX proportional-hazards regression for biliary adverse events development

	Coefficient b	p value	Hazard ratio (HR)	95% CI of HR
Covariate (Model 1)				
Baseline IGF-I/ULN	0.303	**0.009**	1.354	1.079 to 1.699
Mean SRL monthly dose	− 0.009	0.481	0.990	0.964 to 1.017
Octreotide/lanreotide	0.397	0.715	1.487	0.178 to 12.392
Age at baseline	− 0.006	0.565	0.994	0.973 to 1.014
Covariate (Model 2)				
Baseline IGF-I/ULN	0.282	**0.013**	1.354	1.060 to 1.657
Diabetes	− 0.003	0.996	0.996	0.476 to 2.087
Hypertrygliceridemia	− 0.031	0.943	0.969	0.413 to 2.274
Famale gender	− 0.033	0.258	0.717	0.404 to 1.272
Covariate (Model 3)				
Baseline IGF-I/ULN	0.302	**0.011**	1.352	1.073 to 1.700
Baseline glucose	0.002	0.794	1.002	0.984 to 1.021
HbA1c	− 0.019	0.981	0.981	0.927 to 1.038
Baseline GH	− 0.006	0.994	0.994	0.973 to 1.016

Statistically significant values (p < 0.05) are given in bold

*ULN* upper limit of normal, *CI* confidence interval, *HbA1c* glycosylated hemoglobin, *BMI* body mass index, *SRL* Somatostatin receptor ligands, *GH* growth hormone, *IGF*-*I* insulin like growth factor I

### Cholelithiasis or sludge

G_CH_ and G_SL_ comparative data are summarized in Table 3. At last follow-up, disease control was significantly different between these groups, indeed GH (0.89, 0.42–1.65 vs 2.6, 1.9–4.82 ng/mL; p = 0.001) and IGF-I levels (200, 144–26 vs 323, 233–659 ng/mL; p = 0.010) were lower in G_CH_ than in G_SL_, as well as IGF-I/ULN ratio (0.82, 0.52–1.04 vs 1.5, 0.78–2.05; p = 0.005). The fasting glucose levels were higher in G_SL_ (100, 90–115 vs 94, 84–103 mg/dL; p = 0.030) compared to G_CH_, but levels of HbA1c overlapped in the two groups. The triglycerides levels were significantly higher in those who developed gallstones (90.5 ± 28 vs 121 ± 59 mg/dL; p = 0.015) in comparison with G_SL_ while other lipid profile variables were similar in the two groups. Finally, a higher but not significant BMI value was recorded (p = 0.062) in subjects who developed cholelithiasis (26 ± 4 vs 28 ± 6 kg/m^2^), although the obesity rate was similar (17% in G_SL_ and 34% in G_CH_; p = 0.312). Cholelithiasis developed after a longer period (36, 12–144 months) compared to sludge alone (12, 12–120 months), although this difference does not reach statistical significance (p = 0.08). Also, in this case, no difference was detected between treatment with octreotide and lanreotide.

**Table 3:** Features at last follow-up of patients with sludge (G_SL_) or cholelithiasis (G_CH_)

	G_SL_^a^	G_CH_^b^	p value
GH (ng/mL) median IQR	2.6 (1.9–4.8)	0.9 (0.4–1.6)	**0.001**
IGF-I (ng/mL)median IQR	323 (233–659)	200 (144–269)	**0.001**
IGF-I/UNL median IQR	1.5 (0.8–2.1)	0.8 (0.5–1)	**0.005**
Plasma glucose (mg/dL) median IQR	100 (90–115)	94 (84–103)	**0.026**
HbA1c (mmol/mol) median IQR	43 (41–48)	44 (39–47)	NS
Plasma triglycerides (mg/dL) Mean ± SD	90.5 ± 28	121 ± 59	**0.015**
Plasma HDL (mg/dL) Mean ± SD	58 ± 13	57 ± 17	NS
Non-HDL (mg/dL) Mean ± SD	134 ± 37	143 ± 39	NS
LDL (mg/dL) Mean ± SD	116 ± 36	121 ± 32	NS
BMI (kg/m2) Mean ± SD	26 ± 4	28 ± 6	NS
Obesity n. (%)	3 (17)	11 (34)	NS
Follow up on SRL months median (range)	12 (12–120)	36 (12–144)	NS
Octreotide n. (%)	14 (70)	23 (74)	NS
Lanreotide n. (%)	6 (30)	8 (26)	
UDCA therapy n. (%)	17 (74)	23 (70)	NS
Regression on UDCA n. (%)	17 (88)	7 (30)	**<** **0.001**

Statistically significant values (p < 0.05) are given in bold

*GH* growth hormone, *IQR* interquartile range, *SD* standard deviation, *ULN* upper limit of normal, *HbA1c* glycosylated hemoglobin, *BMI* body mass index, *SRL* Somatostatin receptor ligands, *UDCA* ursodeoxycholic acid, *NS* p > 0.05, *HDL* high density lipoproteins, *LDL* low density lipoproteins, *IGF*-*I* insulin like growth factor I

^a^Calculated in G_CH_ at onset of sludge

^b^In G_SL_ at onset of cholelithiasis

### Symptomatic cholelithiasis and cholecystectomy

In our cohort, only five patients underwent cholecystectomy, four in “election” because of symptoms, and one in “urgency” for pancreatitis. Among this group, four patients were obese (BMI > 30 kg/m^2^) and all five were affected by multi-factorial dyslipidaemia, two of these in diet therapy and three on statin. However, none of the patients had diabetes, but two patients had impaired fasting glucose. All five patients were on octreotide LAR (20–30 mg monthly) and biliary symptoms appeared respectively after a median of 48 (12–204) months after the start of SRL therapy. In three patients there had been a previous finding of sludge and microlithiasis, while in two cases cholelithiasis was suddenly symptomatic. Two of the three patients with previous sludge were on UDCA therapy and the symptoms appeared after 36 and 96 months of therapy, respectively.

### Ursodeoxycholic acid treatment

**Fig. 3:**
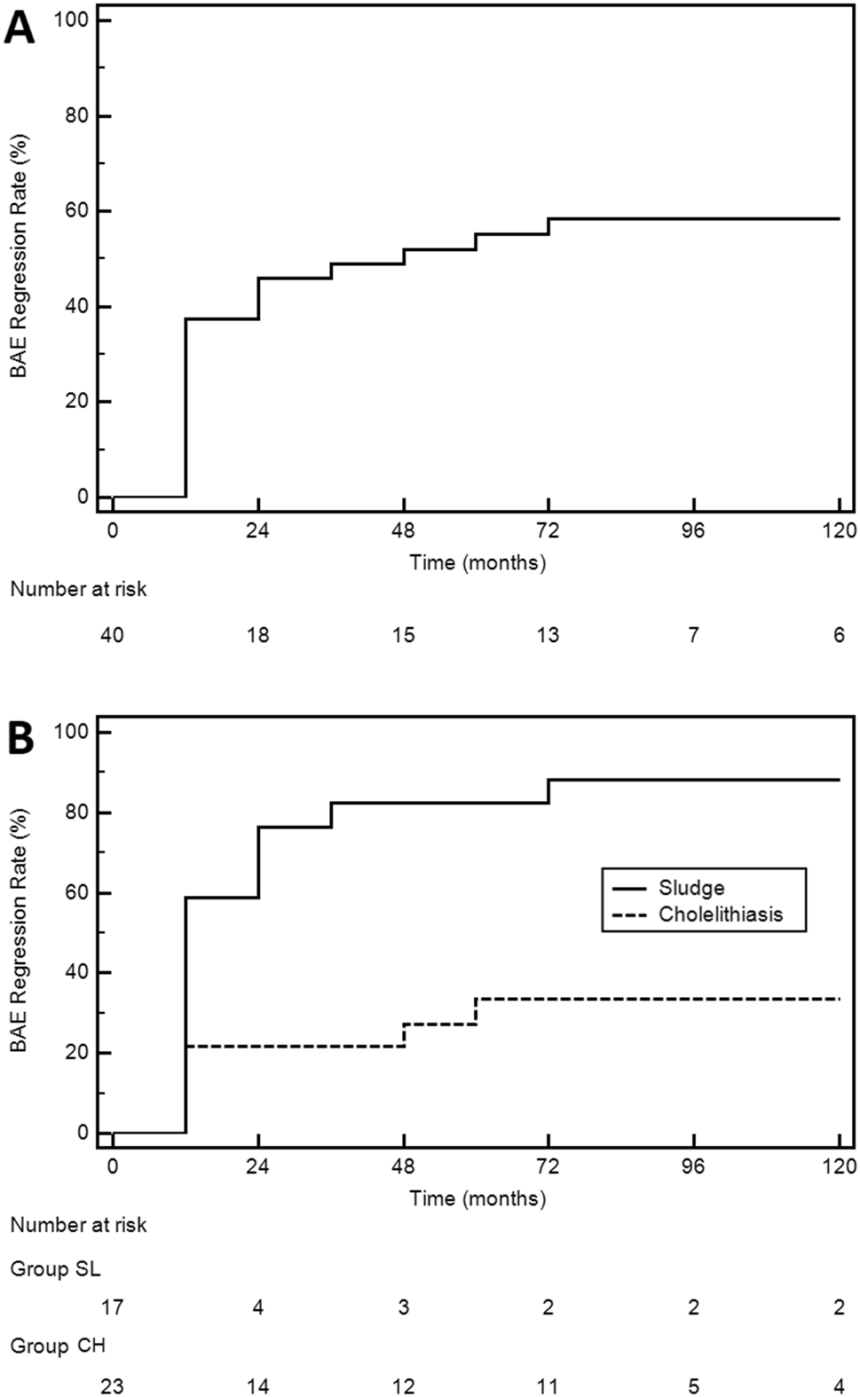
Kaplan–Meier curve for any biliary adverse event (BAE) regression after ursodeoxycholic acid therapy (**a**), and comparison between regression in cholelithiasis (CH) group and only sludge (SL) group (**b**)

In 40/56 subjects (71%) with a biliary complication UDCA treatment was started at the dose of 450 mg per day; 22/40 (55%) had a complete resolution without recurrence over the years, while 18/40 (45%) did not benefit from therapy. In patients with obtained BAE regression, we detected a better metabolic profile. In fact, BMI (p = 0.012), triglycerides (p = 0.044), LDL (p = 0.020), non-HDL cholesterol (p = 0.037) levels, obesity rate were significantly lower (p 0.017), whereas GH (p = 0.005) and IGF-I/ULN (0.058) values were higher in the BAE regression group, if compared to patients without BAE regression (Table 4). The effectiveness of UDCA therapy was higher (p < 0.001) in subjects with only sludge (regression in 88% of cases) compared to those with microlithiasis (regression in 30% of cases). As shown in Fig. 3a, the regression of biliary alterations occurred within 12 months from the start of UDCA treatment in about the 50% of patients. The time dependent analysis confirmed the lower efficacy of therapy in G_CH_ patients (p < 0.001; Fig. 3b). Furthermore, the continuation of the therapy over 6 years does not entail any further benefit. Finally, in the univariate analysis with Cox regression model, cholelithiasis (HR 0.270, 95% CI 0.1084–0.674, p = 0.005), obesity (HR 0.282, 95% CI 0.083–0.955, p = 0.043) and high BMI (HR 0.899, 95% CI 0.818–0.989, p = 0.029) before UDCA starting, confirmed to be negative predictors of treatment effectiveness, while higher GH levels assessed before UDCA were a positive predictor (HR 1.081, CI 1.003–1.166, p = 0.043). However, in the multivariate Cox regression analyses only the presence of cholelithiasis remained a strong predictor of UDCA resistance (Table 5).

**Table 4:** Clinical features before ursodeoxycholic acid starting

	Overall data (n. 40)	No regression (n. 18)	Regression (n. 22)	p value
Age (years, mean ± SD)	52.4 ± 1.4	51.3 ± 13.6	53.4 ± 15.3	NS
Male n. (%)	15 (37.5)	6 (40)	9 (60)	NS
IGF-I (ng/mL) median IQR	235 (182.5–420)	216.5 (151–353)	263 (210– 626)	NS
GH (ng/mL) median IQR	1.9 (0.7–3.8)	0.9 (0.4–2)	3 (1.6–4.5)	**0.005**
IGF-I/ULN median IQR	0.9 (0.7–2.4)	0.8 (0.5–1.1)	1.0 (0.8–1.9)	0.058
Hypertrygliceridemia n. (%)	4 (10)	3 (75)	1 (25)	NS
Trygliceredes (mg/dL) median IQR	97 (80–126)	116 (88.7–134.5)	87 (72.7–113.7)	**0.044**
LDL levels (mg/dL) mean ± SD	123.9 ± 37.7	140.1 ± 33.6	111.6 ± 36.6	**0.020**
HDL levels (mg/dL) mean ± SD	58.3 ± 15.1	54.4 ± 12.4	61.3 ± 16.6	NS
Non-HDL (mg/dL) mean ± SD	143.4 ± 42.3	159.9 ± 39.8	130.9 ± 40.5	**0.037**
GGT (UI/L) median IQR	15 (11–24)	20 (12–32)	15 (11–18)	NS
Diabetes n. (%)	10 (25)	4 (40)	6(60)	NS
Plasma glucose (mg/dL) Mean ± SD	97.8 ± 17.3	97.1 ± 17.3	98.4 ± 17.6	NS
HbA1c (mmol/mol) median IQR	42.5 (40–47)	40.5 (37–46)	43.5 (41–48)	NS
Obesity n (%)	12 (30)	9 (75)	3 (15)	**0.017**
BMI median IQR	26.8 (24–30.4)	30 (26.3–34.6)	26.2 (23.6–27)	**0.012**
Cholelithiasis n. (%)	23 (57.5)	16 (69.6)	7 (30.4)	**<0.001**

Statistically significant values (p < 0.05) are given in bold

*SD* standard deviation, *IQR* interquartile range, *GH* growth hormone, *HbA1c* glycosylated hemoglobin, *GGT* gamma glutamyl transpeptidase, *BMI* body mass index, *CI* confidence interval, *ULN* upper limit of normal, *BAE* biliary adverse events, *NS* p > 0.05, *HDL* high density lipoproteins, *LDL* low density lipoproteins, *IGF-I* insulin like growth factor I

**Table 5:** Multivariate COX proportional-hazards regression for ursodeoxycholic effectiveness

Covariate	Coefficient b	p value	Hazard ratio	95% CI of HR
Model 1				
Cholelithiasis	− 1.038	**0.037**	0.354	0.134 to 0.935
Obesity	− 0.802	NS	0.449	0.122 to 1.652
Model 2				
Cholelithiasis	− 1.031	**0.04**	0.36	0.134 to 0.95
BMI	− 0.067	NS	0.935	0.842 to 1.04
Model 3				
Cholelithiasis	− 1.540	**0.004**	0.214	0.075 to 0.616
GH	0.024	NS	1.024	0.938 to 1.118
Model 4				
Cholelithiasis	− 1.130	**0.018**	0.323	0.127 to 0.822
LDL	− 0.01	NS	0.99	0.977 to 1.002
Model 5				
Cholelithiasis	− 1.241	**0.009**	0.289	0.114 to 0.734
IGF-I/ULN	0.355	NS	1.426	0.856 to 2.376

Statistically significant values (p < 0.05) are given in bold

*CI* confidence interval, *GH* growth hormone, *ULN* upper limit of normal, *BMI* body mass index, *NS* p > 0.05, *IGF-I* insulin like growth factor I, *LDL* low density lipoproteins

### Pasireotide

The 5 patients switched to pasireotide, because of first generation SRL resistance, were excluded from the analysis, due to the very small sample size. In four cases biliary complications occurred: two patients developed sludge, one patient developed sludge and then asymptomatic cholelithiasis and another one developed sludge and then symptomatic microlithiasis. Biliary complications occurred within the first 2 years of treatment and only in one case the duration of previous therapy with first-generation analogs lasted longer than 1 year.

## Discussion

The present study showed as acromegaly activity before starting SRL, sensitivity to SRL and metabolic profile are the main determinants of biliary stone disease in acromegaly population.

At our knowledge, this is the largest longitudinal retrospective study involving acromegaly patients under SRL, attending our tertiary referral Center, in which biliary adverse events were systematically evaluated. Our data confirm that gallbladder alterations represent a common adverse event for acromegaly patients during SRL treatment, particularly within the first 5 years of therapy. Conversely, after 10 years of continuative treatment, the risk of gallbladder sludge and stones development seems to be very low and ultrasound monitoring could be less tight.

In our study, 61.5% of patients developed at least one gallbladder alteration (sludge and/or gallstone); this rate is higher than previously reported (3.6–56%) [1]. Considering that gallstones development is due to a complex interaction among genetic and environmental factors [12], the higher rate of events in our series could be explained by a limited geographical living area, compared to previous studies. We collected yearly abdominal ultrasound, so we could carefully monitor the entire sludge developing process. In fact, biliary sludge is considered a precocious and reversible stage in developing of gallstones [19, 20]. Usually the removal of responsible main factor leads to sludge regression in most of cases [21] and this is confirmed even in acromegaly patients when SRL is withdrawn. However, SRL could be a lifelong therapy so the biliary sludge impairment risk remains consistent over time. In our experience, in all patients developing SRL-induced gallstones, sludge was present previously or at the same time and no case of regression from gallstone to sludge was detected without medical intervention. In addition, we highlighted that the risk of sludge onset is greater in the first years of SRL. This could lead to change the timing of ultrasound follow-up of biliary complications, which could be tighter during the first years and then less frequent. In accordance with Grasso et al. [1], there are no differences between different SRL molecules in inducing biliary stone disease (octreotide or lanreotide) as well as evident correlation to SRL dose. In our study subjects receiving pasireotide were excluded from the analysis for the small sample, but, differently to the data in the Literature, its use appears to be associated with a higher frequency of BSD compared to the first generation analogs [22, 23].

Most of studies in this field did not found an association between acromegaly disease and sludge onset, without concomitant SRL therapy [17]. Conversely, in our study IGF-I and IGF-I/ULN were significant positive predictor of biliary adverse events development. These findings might confirm the hypothesis that a reduced gallbladder emptying and slower bowel transit, associated to supersaturated in cholesterol bile, typical of acromegalic subjects, could promote biliary onset in patients with a more aggressive disease before SRL starting [5, 16].

The debate about the role of metabolic alterations in this field is also open. In general population diabetes and dyslipidaemia were proved to be risk factors for gallstone development [10–12]. However, in the most recent studies, non-HDL cholesterol seems to be the only relevant factor [13]. In acromegaly population, Attanasio et al. found a statistical association between dyslipidemia, obesity and gallstones. In our series neither diabetes nor dyslipidaemia were associated to a high rate of biliary complications [17]. According to Attanasio et al., in our study, sludge-stone transformation would occur more frequently in male population and female gender does not represent a significant risk factor. However, this result is in contrast with the epidemiological data in the general population [12].

A distinct discussion should be done for G_CH_ and G_SL_ groups. To date, no previous studies have investigated the differences between patients who develop only sludge and those who also develop cholelithiasis during SRL therapy. In fact, we found some differences both in terms of acromegaly control and metabolic profile. First of all, GH, IGF-I and IGF-I/ULN values are significantly lower in subjects who developed cholelithiasis. A possible explanation could be the greater affinity of the somatostatin receptors expressed both on pituitary lesion and at intestinal level in well controlled patients with a consequent greater risk of cholelithiasis evolution, principally because of reduction of cholecystokinin release from the small intestine [3] and inhibition of the usual prandial relaxation of the sphincter of Oddi, motility, and emptying of the gallbladder [4].

As metabolic profile is concerned, we highlighted higher blood glucose levels in patients G_SL_. However, the glycosilated hemoglobin levels, which more adequately reflect the glycaemic control, were not statistically different indicating a similar profile in the two groups. We also detected higher triglycerides levels in G_CH_ that could be interpreted as a risk factor for the progression from sludge to gallstones. As we expected, cholelithiasis onset took a longer time than sludge (5 years vs 2 years since the SRL start), confirming the hypothesis of a continuous pathological process in which the occurrence of sludge precedes the formation of stones.

Seventy-one percent of subjects who developed BAE received UDCA, which proved to be effective in 55% of cases, particularly in subjects developing only sludge (88%) rather than cholelithiasis (30%). Our results are consistent with data reported by Guarino et al. [24], in particular the regression of biliary complications occurred after 12 months of UDCA therapy also in our series. From the Kaplan–Meier curve analysis, however, all subjects in which UDCA was effective had a sludge/gallstone regression within 70 months. So, in the absence of side effects, we suggest 5 years UDCA long term treatment even in case of non-regression after the first year [24]. An interesting finding, never described before in acromegaly patients, is the relationship between obesity, BMI and the efficacy of UDCA treatment. In fact, as showed by Cox regression analysis, these two factors significantly reduce the probability of a successful therapy, suggesting that a dose-weight related UDCA therapy and a lifestyle changing approach should be added to the standard clinical practice. Conversely higher IGF-I/ULN and GH levels seemed to promote BAE regression during UDCA, probably because of the lower response to SRL in these patients, as described above.

In our series we confirmed that cholelithiasis is asymptomatic in most cases. In the general population sludge and microlithiasis generally remain asymptomatic with an evolution risk to symptomatic forms of 10% in 5 years and 20% in 20 years. Although the risk of complications in the asymptomatic phase is low, when it becomes symptomatic the risk of acute cholecystitis, chronic cholecystitis, choledocholithiasis, idiopathic pancreatitis, Mirizzi syndrome, porcelain gallbladder and gallbladder carcinoma seem to increase [25]. The small sample of symptomatic patients in our work does not consent an accurate analysis, but all subjects who underwent surgery were obese. Previous study indeed demonstrated obesity as one of main risk factors for biliary complications, mostly in female [17, 26]. In our population, instead, obesity did not prove to be statistically associated to sludge or gallstone developing.

The strength of our study is the long follow-up and the availability of yearly ultrasound evaluation as well as biochemical investigations. Even though our study showed data from a tertiary Center of Neuroendocrinology, our experience could be easily applicable in clinical practice and support clinicians in biliary adverse event management in acromegaly patients. The present study has also some limitations, in particular the retrospective design and the fixed UDCA treatment dose did not allow us to evaluate the efficacy of different doses of UDCA in obese patients.

## Conclusion

Biliary adverse events are frequent in acromegaly patients treated with SRL and it could develop in over half of subjects. Higher IGF-I levels before starting SRL therapy could promote sludge onset. On the other hand, a greater sensitivity to SRL treatment and higher triglycerides levels seem to be associated to a greater probability of gallstone progression, as well as obesity could be a promoting factor for symptomatic disease. In most cases SRL-induced cholelithiasis is asymptomatic and therefore the Guidelines suggest that ultrasound monitoring should not be performed unless the symptoms appear [27]. However, since the lithogenesis is a step process (where the sludge represents its first stage), early diagnosis and the appropriate treatment with UDCA could allow stopping the progression to sludge/microlithiasis and gallstones and reducing the risk of complications. Finally, our findings could open to a tailored approach in preventing, diagnosis and treatment of biliary disease for each acromegaly patient who needs SRL therapy, based on disease activity, SRL sensitivity and metabolic profile.

## Data Availability

The data used to support the findings of this study are available from the corresponding author upon request.
